# Chlorido(η^5^-cyclo­penta­dien­yl)[(4a,4b,8a,9,9a-η)-fluoren­yl](fluorenyl-κ*C* ^9^)zirconium(IV) toluene solvate

**DOI:** 10.1107/S1600536810050816

**Published:** 2010-12-08

**Authors:** Agnieszka Łapczuk-Krygier, Łukasz Ponikiewski, Jerzy Pikies

**Affiliations:** a Chemical Faculty, Gdansk University of Technology, Narutowicza 11/12, Gdansk PL-80233, Poland

## Abstract

In the title compound, [Zr(C_5_H_5_)(C_13_H_9_)_2_Cl]·C_7_H_8_, the Zr^IV^ atom is coordinated by a Cl atom, a cyclo­penta­dienyl (Cp) ligand [Zr–centroid (Cp) = 2.199 (3) Å] and two fluorenyl ligands (Fl) [Zr–centroid (Fl) = 2.273 (2) Å and Zr—CH from fluorenyl = 2.355 (2) Å] in a distorted tetra­gonal geometry. The dihedral angles between the mean planes of the fluorenyl ring systems and the Cp ring are 36.62 (6)° for the η^1^-coordinated fluorenyl and 52.85 (6)° for the η^5^-coordinated fluorenyl,  while the dihedral angle between the mean planes of the two fluorenyl ring systems is 76.18 (7)°.

## Related literature

Unbridged metallocene complexes with fluorenyl ligands constitute precursors of catalysts for homogeneous polymerization of α-olefins, see: Schmid *et al.* (1995) ▶; Alt & Samuel (1998 ▶). Fluorenyl ligands can reduce the stability of complexes, see: Samuel & Setton (1965 ▶). For the preparation of CpZrCl_3_·DME (DME = 1,2-dimethoxyethane), see: Lund & Livinghouse (1990 ▶).
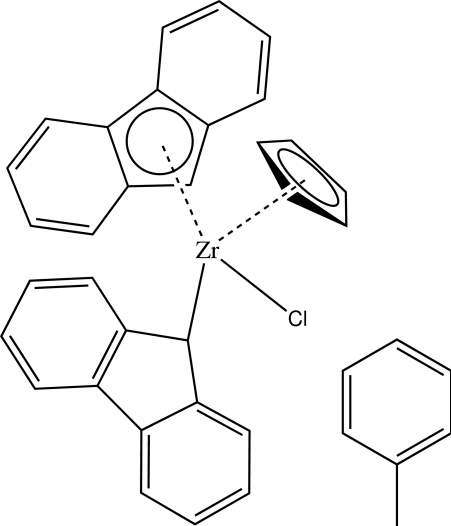


## Experimental

### 

#### Crystal data

[Zr(C_5_H_5_)(C_13_H_9_)_2_Cl]·C_7_H_8_
*M* *_r_* = 614.3
Triclinic, 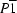

*a* = 9.3091 (4) Å
*b* = 10.7937 (4) Å
*c* = 15.1219 (8) Å
α = 77.231 (4)°
β = 81.966 (4)°
γ = 74.135 (4)°
*V* = 1420.31 (11) Å^3^
*Z* = 2
Mo *K*α radiation
μ = 0.51 mm^−1^
*T* = 150 K
0.35 × 0.16 × 0.07 mm

#### Data collection

Oxford Diffraction Xcalibur Sapphire2 diffractometer
Absorption correction: analytical (*CrysAlis PRO*; Oxford Diffraction, 2010 ▶) *T*_min_ = 0.894, *T*_max_ = 0.97
8923 measured reflections
5572 independent reflections
4680 reflections with *I* > 2σ(*I*)
*R*_int_ = 0.022

#### Refinement

*R*[*F* ^2^ > 2σ(*F* ^2^)] = 0.031
*wR*(*F* ^2^) = 0.076
*S* = 1.05
5572 reflections
362 parameters
H-atom parameters constrained
Δρ_max_ = 0.59 e Å^−3^
Δρ_min_ = −0.33 e Å^−3^

### 

Data collection: *CrysAlis CCD* (Oxford Diffraction, 2009 ▶); cell refinement: *CrysAlis RED* (Oxford Diffraction, 2009 ▶); data reduction: *CrysAlis RED* (Oxford Diffraction, 2009 ▶); program(s) used to solve structure: *SHELXS97* (Sheldrick, 2008 ▶); program(s) used to refine structure: *SHELXL97* (Sheldrick, 2008 ▶); molecular graphics: *ORTEP-3* (Farrugia, 1997 ▶); software used to prepare material for publication: *WinGX32* (Farrugia, 1999 ▶).

## Supplementary Material

Crystal structure: contains datablocks global, I. DOI: 10.1107/S1600536810050816/kp2294sup1.cif

Structure factors: contains datablocks I. DOI: 10.1107/S1600536810050816/kp2294Isup2.hkl

Additional supplementary materials:  crystallographic information; 3D view; checkCIF report

## Figures and Tables

**Table 1:** Selected bond lengths (Å)

Cl1—Zr1	2.4537 (5)
Zr1—C1	2.521 (2)
Zr1—C2	2.515 (2)
Zr1—C3	2.490 (2)
Zr1—C4	2.467 (2)
Zr1—C5	2.499 (2)
Zr1—C6	2.355 (2)
Zr1—C19	2.468 (2)
Zr1—C28	2.6434 (19)
Zr1—C29	2.617 (2)
Zr1—C30	2.601 (2)
Zr1—C31	2.565 (2)

**Fig. 1.**
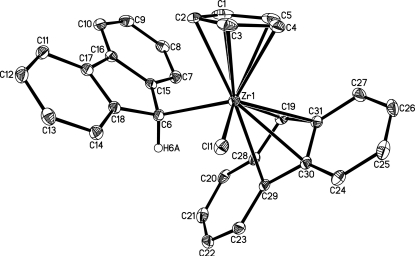
The molecular structure of the title molecule with the atom-numbering. Displacement ellipsoids are drawn at the 30% probability level. The H atoms bonded to C atoms (except H6A) were omitted for clarity.

## supplementary crystallographic information

### Comment

The unbridged metallocene complexes with fluorenyl ligand constitute precursors of catalysts for homogeneous polymerization of α-olefins (Scmid *et al.* 1995; Alt *et al.* 1998). The fluorenyl ligands facile changes in hapticity η^5^→η^1^ (ring-slippage). This property influences the catalytic activity of this type of compounds, however it also hampers to syntheses and lowers the stability of the complexes, for example Flu_2_ZrCl_2_ is stable in donor solvent (THF) for only a short time (Samuel *et al.* 1965).

The structure exhibits an η^5 ^,η^1^ fluorenyl coordination to the zirconium mononuclear centre, completing the coordinations sphere of a chloride atom and cyclopentadienyl ligand (Fig. 1 and Table 1). The fluorenyl groups are not exactly planar, r.m.s. deviations of a best least-squares plane of the fluorenyl units are: for η^1^– coordinated is 0.042 (6) Å and for η^5^– coordinated is 0.132 (6) Å (the values were found for carbon atoms). The dihedral angles between the mean planes of the fluorenyl ring systems and the cyclopentadienyl ring are: η^1^– coordinated fluorenyl and Cp 36.62 (6)° and η^5^– coordinated fluorenyl and Cp 52.85 (6)°, however the dihedral angle between the mean planes of the two fluorenyl system ring system is 103.82 (7)°.

### Experimental

All reactions and manipulations were carried aot under an atmosphere of ultra-high purified argon employing standard Schlenk techniques. Solvents were purified, dried and distilled prior to use from dark blue potassium or sodium diphenyl ketyl solution.

CpZrCl_3_.DME was prepared according to the literature (Lund *et al.* 1990). Fluorene is commercial product and was used without further purification.

A solution of fluorene in Et_2_O was treated with *n*-BuLi (1,6M in hexane). After the evolution of gas completes, an equimolar amount of CpZrCl_3_.DME was added. The mixture was stirred for 2 h. The solvent was removed in vaccum. The residue was extracted with toluene and the suspension was filtered through magnesium sulfate. The filtrated was concetrated and crystallised at 251 K (Schmid *et al.* 1995).

### Refinement

All H atoms were fixed geometrically and treated as riding with C—H = 0.95 Å (aromatic), 0.98 Å (methyl) and 1.00 Å (methine) with *U*_iso_(H) = 1.2 Ueq (aromatic, methine, methylene) and *U*_iso_(H) = 1.5Ueq (methyl).

### Crystal data

**Table:**

[Zr(C_5_H_5_)(C_13_H_9_)_2_Cl]·C_7_H_8_	*Z* = 2
*M**_r_* = 614.3	*F*(000) = 632
Triclinic, *P*1	*D*_x_ = 1.436 Mg m^−^^3^
Hall symbol: -P 1	Mo *K*α radiation, λ = 0.71073 Å
*a* = 9.3091 (4) Å	Cell parameters from 6309 reflections
*b* = 10.7937 (4) Å	θ = 2.6–28.7°
*c* = 15.1219 (8) Å	µ = 0.51 mm^−^^1^
α = 77.231 (4)°	*T* = 150 K
β = 81.966 (4)°	Block, yellow
γ = 74.135 (4)°	0.35 × 0.16 × 0.07 mm
*V* = 1420.31 (11) Å^3^	

### Data collection

**Table:**

Oxford Diffraction Xcalibur Sapphire2 large Be window diffractometer	5572 independent reflections
graphite	4680 reflections with *I* > 2σ(*I*)
Detector resolution: 8.1883 pixels mm^-1^	*R*_int_ = 0.022
ω scans	θ_max_ = 26°, θ_min_ = 2.6°
Absorption correction: analytical (*CrysAlis PRO*; Oxford Diffraction, 2010)	*h* = −10→11
*T*_min_ = 0.894, *T*_max_ = 0.97	*k* = −13→12
8923 measured reflections	*l* = −13→18

### Refinement

**Table:**

Refinement on *F*^2^	Primary atom site location: structure-invariant direct methods
Least-squares matrix: full	Secondary atom site location: difference Fourier map
*R*[*F*^2^ > 2σ(*F*^2^)] = 0.031	Hydrogen site location: inferred from neighbouring sites
*wR*(*F*^2^) = 0.076	H-atom parameters constrained
*S* = 1.05	*w* = 1/[σ^2^(*F*_o_^2^) + (0.0473*P*)^2^] where *P* = (*F*_o_^2^ + 2*F*_c_^2^)/3
5572 reflections	(Δ/σ)_max_ = 0.001
362 parameters	Δρ_max_ = 0.59 e Å^−^^3^
0 restraints	Δρ_min_ = −0.33 e Å^−^^3^

### Special details

**Table:**

Experimental. CrysAlisPro, Oxford Diffraction Ltd., Version 1.171.33.66 (release 28-04-2010 CrysAlis171 .NET) (compiled Apr 28 2010,14:27:37) Analytical numeric absorption correction using a multifaceted crystal model based on expressions derived by R.C. Clark & J.S. (Clark, R. C. & Reid, J. S. (1995). Acta Cryst. A51, 887-897)
Geometry. All s.u.'s (except the s.u. in the dihedral angle between two l.s. planes) are estimated using the full covariance matrix. The cell s.u.'s are taken into account individually in the estimation of s.u.'s in distances, angles and torsion angles; correlations between s.u.'s in cell parameters are only used when they are defined by crystal symmetry. An approximate (isotropic) treatment of cell s.u.'s is used for estimating s.u.'s involving l.s. planes.
Refinement. Refinement of *F*^2^ against ALL reflections. The weighted *R*-factor *wR* and goodness of fit *S* are based on *F*^2^, conventional *R*-factors *R* are based on *F*, with *F* set to zero for negative *F*^2^. The threshold expression of *F*^2^ > 2σ(*F*^2^) is used only for calculating *R*-factors(gt) *etc*. and is not relevant to the choice of reflections for refinement. *R*-factors based on *F*^2^ are statistically about twice as large as those based on *F*, and *R*- factors based on ALL data will be even larger.

### Fractional atomic coordinates and isotropic or equivalent isotropic displacement parameters (Å^2^)

**Table:**

	*x*	*y*	*z*	*U*_iso_*/*U*_eq_	
Cl1	0.63228 (6)	0.47339 (5)	0.60738 (4)	0.02355 (13)	
Zr1	0.60062 (2)	0.637778 (18)	0.702371 (14)	0.01694 (7)	
C1	0.6240 (3)	0.6138 (3)	0.86975 (16)	0.0380 (6)	
H1A	0.5588	0.6722	0.9104	0.046*	
C2	0.6023 (3)	0.4959 (3)	0.85859 (17)	0.0372 (7)	
H2A	0.5201	0.4548	0.8903	0.045*	
C3	0.7277 (3)	0.4361 (2)	0.80616 (17)	0.0325 (6)	
H3A	0.7511	0.3445	0.795	0.039*	
C4	0.8250 (3)	0.5197 (2)	0.78403 (16)	0.0301 (5)	
H4A	0.9301	0.4969	0.7556	0.036*	
C5	0.7600 (3)	0.6282 (2)	0.82490 (16)	0.0328 (6)	
H5A	0.81	0.6977	0.8287	0.039*	
C6	0.3404 (2)	0.6697 (2)	0.73870 (14)	0.0191 (4)	
H6A	0.2874	0.7264	0.6852	0.023*	
C7	0.2992 (2)	0.8535 (2)	0.83522 (16)	0.0245 (5)	
H7A	0.3413	0.9115	0.7887	0.029*	
C8	0.2488 (3)	0.8869 (2)	0.91958 (16)	0.0280 (5)	
H8A	0.2561	0.9685	0.93	0.034*	
C9	0.1876 (3)	0.8034 (2)	0.98953 (16)	0.0302 (5)	
H9A	0.1543	0.8281	1.0469	0.036*	
C10	0.1755 (2)	0.6844 (2)	0.97514 (15)	0.0273 (5)	
H10A	0.1344	0.6268	1.0225	0.033*	
C11	0.1724 (2)	0.4232 (2)	0.89921 (16)	0.0259 (5)	
H11A	0.1333	0.4136	0.9611	0.031*	
C12	0.1788 (2)	0.3280 (2)	0.84944 (17)	0.0286 (5)	
H12A	0.1444	0.2523	0.8775	0.034*	
C13	0.2356 (2)	0.3428 (2)	0.75838 (17)	0.0273 (5)	
H13A	0.2368	0.2779	0.7247	0.033*	
C14	0.2905 (2)	0.4511 (2)	0.71600 (16)	0.0239 (5)	
H14A	0.3297	0.4598	0.6541	0.029*	
C15	0.2880 (2)	0.7342 (2)	0.81871 (15)	0.0203 (4)	
C16	0.2244 (2)	0.6503 (2)	0.89033 (15)	0.0220 (5)	
C17	0.2242 (2)	0.5333 (2)	0.85688 (15)	0.0222 (5)	
C18	0.2872 (2)	0.5465 (2)	0.76560 (15)	0.0210 (5)	
C19	0.6192 (2)	0.86760 (19)	0.66605 (15)	0.0224 (5)	
H19A	0.6112	0.9236	0.712	0.027*	
C20	0.3519 (2)	0.9527 (2)	0.60587 (16)	0.0248 (5)	
H20A	0.3105	1.0121	0.6461	0.03*	
C21	0.2667 (3)	0.9381 (2)	0.54421 (17)	0.0298 (5)	
H21A	0.1659	0.9889	0.5416	0.036*	
C22	0.3247 (3)	0.8497 (2)	0.48431 (16)	0.0287 (5)	
H22A	0.2611	0.839	0.4441	0.034*	
C23	0.4706 (2)	0.7793 (2)	0.48319 (15)	0.0235 (5)	
H23A	0.5094	0.7205	0.4422	0.028*	
C24	0.8395 (2)	0.6781 (2)	0.49803 (15)	0.0241 (5)	
H24A	0.8172	0.6418	0.4513	0.029*	
C25	0.9851 (3)	0.6621 (2)	0.51474 (18)	0.0312 (6)	
H25A	1.0645	0.6159	0.4784	0.037*	
C26	1.0185 (3)	0.7137 (2)	0.58524 (18)	0.0326 (6)	
H26A	1.1204	0.7021	0.5949	0.039*	
C27	0.9089 (3)	0.7794 (2)	0.63988 (17)	0.0282 (5)	
H27A	0.9339	0.811	0.6881	0.034*	
C28	0.5030 (2)	0.87790 (19)	0.60909 (15)	0.0200 (4)	
C29	0.5631 (2)	0.79551 (19)	0.54418 (14)	0.0185 (4)	
C30	0.7233 (2)	0.74927 (19)	0.55153 (14)	0.0190 (4)	
C31	0.7566 (2)	0.80007 (19)	0.62379 (14)	0.0203 (4)	
C32	0.8254 (3)	1.0698 (2)	0.77764 (16)	0.0279 (5)	
C33	0.6720 (3)	1.1262 (2)	0.77990 (17)	0.0301 (5)	
H33A	0.6339	1.2032	0.737	0.036*	
C34	0.5739 (3)	1.0711 (2)	0.84437 (19)	0.0356 (6)	
H34A	0.4694	1.1113	0.8459	0.043*	
C35	0.6277 (3)	0.9582 (2)	0.90619 (18)	0.0363 (6)	
H35A	0.5604	0.9194	0.9495	0.044*	
C36	0.7790 (3)	0.9024 (2)	0.90458 (17)	0.0352 (6)	
H36A	0.8165	0.8254	0.9476	0.042*	
C37	0.8768 (3)	0.9567 (2)	0.84148 (17)	0.0318 (6)	
H37A	0.9812	0.9164	0.8413	0.038*	
C38	0.9341 (3)	1.1292 (3)	0.7093 (2)	0.0481 (7)	
H38A	1.0108	1.144	0.7412	0.072*	
H38B	0.8802	1.213	0.6747	0.072*	
H38C	0.9821	1.0691	0.6676	0.072*	

### Atomic displacement parameters (Å^2^)

**Table:**

	*U*^11^	*U*^22^	*U*^33^	*U*^12^	*U*^13^	*U*^23^
Cl1	0.0294 (3)	0.0187 (3)	0.0243 (3)	−0.0091 (2)	0.0016 (2)	−0.0063 (2)
Zr1	0.01916 (11)	0.01453 (11)	0.01689 (12)	−0.00478 (8)	−0.00162 (8)	−0.00174 (8)
C1	0.0457 (15)	0.0388 (14)	0.0186 (12)	0.0117 (13)	−0.0128 (11)	−0.0042 (11)
C2	0.0289 (13)	0.0523 (16)	0.0242 (13)	−0.0176 (12)	−0.0132 (11)	0.0214 (12)
C3	0.0466 (15)	0.0167 (11)	0.0339 (14)	−0.0034 (11)	−0.0220 (12)	0.0015 (10)
C4	0.0216 (11)	0.0386 (13)	0.0254 (13)	−0.0013 (10)	−0.0077 (10)	−0.0007 (10)
C5	0.0491 (15)	0.0287 (12)	0.0262 (13)	−0.0144 (12)	−0.0207 (12)	−0.0002 (10)
C6	0.0175 (10)	0.0193 (10)	0.0193 (11)	−0.0051 (9)	−0.0007 (8)	−0.0012 (8)
C7	0.0220 (11)	0.0239 (11)	0.0265 (12)	−0.0056 (9)	−0.0016 (9)	−0.0036 (9)
C8	0.0292 (12)	0.0284 (12)	0.0280 (13)	−0.0052 (10)	−0.0063 (10)	−0.0085 (10)
C9	0.0310 (13)	0.0362 (13)	0.0225 (12)	−0.0028 (11)	−0.0046 (10)	−0.0092 (10)
C10	0.0234 (11)	0.0345 (13)	0.0218 (12)	−0.0067 (10)	−0.0022 (9)	−0.0014 (10)
C11	0.0215 (11)	0.0263 (12)	0.0258 (12)	−0.0062 (9)	−0.0028 (9)	0.0042 (10)
C12	0.0249 (11)	0.0232 (11)	0.0362 (14)	−0.0096 (10)	−0.0053 (10)	0.0034 (10)
C13	0.0247 (11)	0.0213 (11)	0.0368 (14)	−0.0060 (10)	−0.0050 (10)	−0.0060 (10)
C14	0.0219 (11)	0.0243 (11)	0.0250 (12)	−0.0059 (9)	−0.0022 (9)	−0.0032 (9)
C15	0.0152 (10)	0.0212 (10)	0.0226 (11)	−0.0020 (9)	−0.0059 (9)	−0.0010 (9)
C16	0.0178 (10)	0.0256 (11)	0.0211 (11)	−0.0038 (9)	−0.0043 (9)	−0.0013 (9)
C17	0.0174 (10)	0.0232 (11)	0.0239 (12)	−0.0037 (9)	−0.0039 (9)	−0.0004 (9)
C18	0.0171 (10)	0.0206 (10)	0.0239 (12)	−0.0042 (9)	−0.0052 (9)	−0.0002 (9)
C19	0.0312 (12)	0.0148 (10)	0.0226 (12)	−0.0083 (9)	−0.0019 (9)	−0.0034 (9)
C20	0.0271 (11)	0.0163 (10)	0.0244 (12)	−0.0031 (9)	0.0050 (9)	0.0022 (9)
C21	0.0212 (11)	0.0235 (11)	0.0355 (14)	−0.0036 (10)	−0.0020 (10)	0.0103 (10)
C22	0.0260 (12)	0.0322 (13)	0.0279 (13)	−0.0142 (10)	−0.0083 (10)	0.0062 (10)
C23	0.0292 (12)	0.0224 (11)	0.0201 (11)	−0.0112 (10)	−0.0038 (9)	0.0004 (9)
C24	0.0281 (11)	0.0201 (11)	0.0222 (12)	−0.0071 (9)	0.0029 (9)	−0.0022 (9)
C25	0.0239 (12)	0.0235 (12)	0.0387 (14)	−0.0042 (10)	0.0068 (10)	0.0018 (10)
C26	0.0204 (11)	0.0300 (12)	0.0439 (15)	−0.0125 (10)	−0.0059 (11)	0.0101 (11)
C27	0.0280 (12)	0.0269 (12)	0.0320 (13)	−0.0145 (10)	−0.0106 (10)	0.0038 (10)
C28	0.0238 (11)	0.0130 (9)	0.0217 (11)	−0.0068 (9)	0.0001 (9)	0.0012 (8)
C29	0.0209 (10)	0.0160 (10)	0.0173 (11)	−0.0073 (8)	−0.0005 (8)	0.0024 (8)
C30	0.0214 (10)	0.0147 (10)	0.0204 (11)	−0.0079 (8)	−0.0006 (8)	0.0015 (8)
C31	0.0253 (11)	0.0168 (10)	0.0207 (11)	−0.0111 (9)	−0.0031 (9)	0.0007 (8)
C32	0.0325 (12)	0.0286 (12)	0.0262 (13)	−0.0101 (10)	−0.0011 (10)	−0.0103 (10)
C33	0.0387 (14)	0.0213 (11)	0.0325 (14)	−0.0042 (10)	−0.0100 (11)	−0.0093 (10)
C34	0.0261 (12)	0.0378 (14)	0.0484 (17)	−0.0069 (11)	−0.0005 (11)	−0.0232 (12)
C35	0.0465 (15)	0.0375 (14)	0.0322 (14)	−0.0217 (12)	0.0089 (12)	−0.0155 (11)
C36	0.0528 (16)	0.0246 (12)	0.0286 (14)	−0.0077 (12)	−0.0066 (12)	−0.0066 (10)
C37	0.0304 (12)	0.0284 (12)	0.0355 (14)	−0.0001 (11)	−0.0058 (11)	−0.0114 (11)
C38	0.0502 (17)	0.0554 (17)	0.0418 (17)	−0.0236 (15)	0.0075 (14)	−0.0098 (14)

### Geometric parameters (Å, °)

**Table:**

Cl1—Zr1	2.4537 (5)	C15—C16	1.422 (3)
Zr1—C1	2.521 (2)	C16—C17	1.462 (3)
Zr1—C2	2.515 (2)	C17—C18	1.415 (3)
Zr1—C3	2.490 (2)	C19—C31	1.430 (3)
Zr1—C4	2.467 (2)	C19—C28	1.441 (3)
Zr1—C5	2.499 (2)	C19—H19A	1
Zr1—C6	2.355 (2)	C20—C21	1.364 (3)
Zr1—C19	2.468 (2)	C20—C28	1.419 (3)
Zr1—C28	2.6434 (19)	C20—H20A	0.95
Zr1—C29	2.617 (2)	C21—C22	1.409 (4)
Zr1—C30	2.601 (2)	C21—H21A	0.95
Zr1—C31	2.565 (2)	C22—C23	1.363 (3)
C1—C5	1.382 (4)	C22—H22A	0.95
C1—C2	1.391 (4)	C23—C29	1.413 (3)
C1—H1A	1	C23—H23A	0.95
C2—C3	1.401 (4)	C24—C25	1.372 (3)
C2—H2A	1	C24—C30	1.411 (3)
C3—C4	1.405 (3)	C24—H24A	0.95
C3—H3A	1	C25—C26	1.411 (4)
C4—C5	1.396 (3)	C25—H25A	0.95
C4—H4A	1	C26—C27	1.362 (4)
C5—H5A	1	C26—H26A	0.95
C6—C15	1.486 (3)	C27—C31	1.421 (3)
C6—C18	1.497 (3)	C27—H27A	0.95
C6—H6A	1	C28—C29	1.424 (3)
C7—C8	1.386 (3)	C29—C30	1.449 (3)
C7—C15	1.398 (3)	C30—C31	1.430 (3)
C7—H7A	0.95	C32—C33	1.389 (3)
C8—C9	1.395 (3)	C32—C37	1.393 (3)
C8—H8A	0.95	C32—C38	1.507 (4)
C9—C10	1.386 (3)	C33—C34	1.390 (4)
C9—H9A	0.95	C33—H33A	0.95
C10—C16	1.393 (3)	C34—C35	1.379 (4)
C10—H10A	0.95	C34—H34A	0.95
C11—C12	1.386 (3)	C35—C36	1.371 (4)
C11—C17	1.395 (3)	C35—H35A	0.95
C11—H11A	0.95	C36—C37	1.372 (4)
C12—C13	1.395 (3)	C36—H36A	0.95
C12—H12A	0.95	C37—H37A	0.95
C13—C14	1.394 (3)	C38—H38A	0.98
C13—H13A	0.95	C38—H38B	0.98
C14—C18	1.394 (3)	C38—H38C	0.98
C14—H14A	0.95		
			
C6—Zr1—Cl1	97.23 (5)	C10—C9—C8	119.9 (2)
C6—Zr1—C4	134.90 (8)	C10—C9—H9A	120.1
Cl1—Zr1—C4	94.36 (6)	C8—C9—H9A	120.1
C6—Zr1—C19	100.25 (7)	C9—C10—C16	119.1 (2)
Cl1—Zr1—C19	132.29 (5)	C9—C10—H10A	120.4
C4—Zr1—C19	103.51 (8)	C16—C10—H10A	120.4
C6—Zr1—C3	107.72 (8)	C12—C11—C17	118.9 (2)
Cl1—Zr1—C3	79.54 (6)	C12—C11—H11A	120.6
C4—Zr1—C3	32.94 (8)	C17—C11—H11A	120.6
C19—Zr1—C3	134.22 (7)	C11—C12—C13	120.4 (2)
C6—Zr1—C5	118.29 (8)	C11—C12—H12A	119.8
Cl1—Zr1—C5	126.93 (6)	C13—C12—H12A	119.8
C4—Zr1—C5	32.65 (8)	C14—C13—C12	121.2 (2)
C19—Zr1—C5	81.20 (7)	C14—C13—H13A	119.4
C3—Zr1—C5	53.79 (8)	C12—C13—H13A	119.4
C6—Zr1—C2	80.83 (8)	C18—C14—C13	119.2 (2)
Cl1—Zr1—C2	100.73 (7)	C18—C14—H14A	120.4
C4—Zr1—C2	54.18 (8)	C13—C14—H14A	120.4
C19—Zr1—C2	125.66 (9)	C7—C15—C16	118.1 (2)
C3—Zr1—C2	32.51 (9)	C7—C15—C6	131.6 (2)
C5—Zr1—C2	53.37 (8)	C16—C15—C6	110.13 (18)
C6—Zr1—C1	87.22 (8)	C10—C16—C15	121.5 (2)
Cl1—Zr1—C1	131.38 (6)	C10—C16—C17	130.5 (2)
C4—Zr1—C1	53.73 (8)	C15—C16—C17	107.99 (19)
C19—Zr1—C1	93.64 (8)	C11—C17—C18	121.1 (2)
C3—Zr1—C1	53.45 (8)	C11—C17—C16	130.9 (2)
C5—Zr1—C1	31.95 (9)	C18—C17—C16	108.07 (18)
C2—Zr1—C1	32.06 (9)	C14—C18—C17	119.29 (19)
C6—Zr1—C31	130.91 (7)	C14—C18—C6	130.6 (2)
Cl1—Zr1—C31	108.22 (5)	C17—C18—C6	110.08 (19)
C4—Zr1—C31	85.07 (7)	C31—C19—C28	106.77 (19)
C19—Zr1—C31	32.94 (7)	C31—C19—Zr1	77.25 (11)
C3—Zr1—C31	117.58 (8)	C28—C19—Zr1	80.45 (12)
C5—Zr1—C31	78.16 (8)	C31—C19—H19A	125
C2—Zr1—C31	131.50 (7)	C28—C19—H19A	125
C1—Zr1—C31	104.49 (8)	Zr1—C19—H19A	125
C6—Zr1—C30	124.42 (7)	C21—C20—C28	119.2 (2)
Cl1—Zr1—C30	79.07 (5)	C21—C20—H20A	120.4
C4—Zr1—C30	100.54 (7)	C28—C20—H20A	120.4
C19—Zr1—C30	54.50 (7)	C20—C21—C22	121.6 (2)
C3—Zr1—C30	125.39 (8)	C20—C21—H21A	119.2
C5—Zr1—C30	106.38 (8)	C22—C21—H21A	119.2
C2—Zr1—C30	154.72 (7)	C23—C22—C21	121.0 (2)
C1—Zr1—C30	135.89 (8)	C23—C22—H22A	119.5
C31—Zr1—C30	32.12 (7)	C21—C22—H22A	119.5
C6—Zr1—C29	92.19 (7)	C22—C23—C29	118.7 (2)
Cl1—Zr1—C29	81.15 (5)	C22—C23—H23A	120.7
C4—Zr1—C29	132.72 (7)	C29—C23—H23A	120.7
C19—Zr1—C29	54.28 (7)	C25—C24—C30	118.8 (2)
C3—Zr1—C29	153.75 (8)	C25—C24—H24A	120.6
C5—Zr1—C29	130.64 (7)	C30—C24—H24A	120.6
C2—Zr1—C29	172.92 (7)	C24—C25—C26	120.8 (2)
C1—Zr1—C29	147.29 (8)	C24—C25—H25A	119.6
C31—Zr1—C29	53.15 (6)	C26—C25—H25A	119.6
C30—Zr1—C29	32.23 (6)	C27—C26—C25	121.9 (2)
C6—Zr1—C28	79.51 (7)	C27—C26—H26A	119
Cl1—Zr1—C28	111.11 (5)	C25—C26—H26A	119
C4—Zr1—C28	135.02 (7)	C26—C27—C31	119.1 (2)
C19—Zr1—C28	32.52 (7)	C26—C27—H27A	120.5
C3—Zr1—C28	166.71 (7)	C31—C27—H27A	120.5
C5—Zr1—C28	113.06 (7)	C20—C28—C29	118.6 (2)
C2—Zr1—C28	144.26 (8)	C20—C28—C19	133.0 (2)
C1—Zr1—C28	117.27 (8)	C29—C28—C19	108.38 (18)
C31—Zr1—C28	52.49 (6)	C20—C28—Zr1	126.77 (14)
C30—Zr1—C28	52.44 (6)	C29—C28—Zr1	73.29 (11)
C29—Zr1—C28	31.41 (7)	C19—C28—Zr1	67.03 (11)
C5—C1—C2	108.6 (2)	C23—C29—C28	120.66 (19)
C5—C1—Zr1	73.16 (14)	C23—C29—C30	131.6 (2)
C2—C1—Zr1	73.72 (14)	C28—C29—C30	107.53 (18)
C5—C1—H1A	125.4	C23—C29—Zr1	120.95 (14)
C2—C1—H1A	125.4	C28—C29—Zr1	75.30 (11)
Zr1—C1—H1A	125.4	C30—C29—Zr1	73.25 (11)
C1—C2—C3	107.7 (2)	C24—C30—C31	120.6 (2)
C1—C2—Zr1	74.21 (13)	C24—C30—C29	131.9 (2)
C3—C2—Zr1	72.78 (13)	C31—C30—C29	107.33 (19)
C1—C2—H2A	125.9	C24—C30—Zr1	122.46 (13)
C3—C2—H2A	125.9	C31—C30—Zr1	72.55 (12)
Zr1—C2—H2A	125.9	C29—C30—Zr1	74.51 (11)
C2—C3—C4	107.9 (2)	C27—C31—C30	118.9 (2)
C2—C3—Zr1	74.71 (12)	C27—C31—C19	132.4 (2)
C4—C3—Zr1	72.61 (12)	C30—C31—C19	108.75 (18)
C2—C3—H3A	125.7	C27—C31—Zr1	122.10 (14)
C4—C3—H3A	125.7	C30—C31—Zr1	75.33 (12)
Zr1—C3—H3A	125.7	C19—C31—Zr1	69.81 (11)
C5—C4—C3	107.3 (2)	C33—C32—C37	117.9 (2)
C5—C4—Zr1	74.96 (13)	C33—C32—C38	121.5 (2)
C3—C4—Zr1	74.45 (12)	C37—C32—C38	120.5 (2)
C5—C4—H4A	125.8	C32—C33—C34	120.6 (2)
C3—C4—H4A	125.8	C32—C33—H33A	119.7
Zr1—C4—H4A	125.8	C34—C33—H33A	119.7
C1—C5—C4	108.5 (2)	C35—C34—C33	120.3 (2)
C1—C5—Zr1	74.89 (14)	C35—C34—H34A	119.9
C4—C5—Zr1	72.39 (13)	C33—C34—H34A	119.9
C1—C5—H5A	125.5	C36—C35—C34	119.4 (2)
C4—C5—H5A	125.5	C36—C35—H35A	120.3
Zr1—C5—H5A	125.5	C34—C35—H35A	120.3
C15—C6—C18	103.35 (17)	C35—C36—C37	120.7 (2)
C15—C6—Zr1	111.95 (13)	C35—C36—H36A	119.6
C18—C6—Zr1	115.03 (13)	C37—C36—H36A	119.6
C15—C6—H6A	108.8	C36—C37—C32	121.1 (2)
C18—C6—H6A	108.8	C36—C37—H37A	119.5
Zr1—C6—H6A	108.8	C32—C37—H37A	119.5
C8—C7—C15	119.8 (2)	C32—C38—H38A	109.5
C8—C7—H7A	120.1	C32—C38—H38B	109.5
C15—C7—H7A	120.1	H38A—C38—H38B	109.5
C7—C8—C9	121.5 (2)	C32—C38—H38C	109.5
C7—C8—H8A	119.2	H38A—C38—H38C	109.5
C9—C8—H8A	119.2	H38B—C38—H38C	109.5
			
C6—Zr1—C1—C5	−167.02 (15)	C1—Zr1—C19—C28	−138.95 (13)
Cl1—Zr1—C1—C5	95.79 (15)	C31—Zr1—C19—C28	109.75 (18)
C4—Zr1—C1—C5	37.18 (14)	C30—Zr1—C19—C28	74.09 (13)
C19—Zr1—C1—C5	−66.94 (15)	C29—Zr1—C19—C28	34.14 (11)
C3—Zr1—C1—C5	78.42 (16)	C28—C20—C21—C22	0.7 (3)
C2—Zr1—C1—C5	115.8 (2)	C20—C21—C22—C23	−2.9 (3)
C31—Zr1—C1—C5	−35.38 (16)	C21—C22—C23—C29	0.6 (3)
C30—Zr1—C1—C5	−27.30 (19)	C30—C24—C25—C26	1.3 (3)
C29—Zr1—C1—C5	−77.34 (19)	C24—C25—C26—C27	0.7 (3)
C28—Zr1—C1—C5	−90.34 (15)	C25—C26—C27—C31	−1.8 (3)
C6—Zr1—C1—C2	77.13 (15)	C21—C20—C28—C29	3.5 (3)
Cl1—Zr1—C1—C2	−20.06 (19)	C21—C20—C28—C19	−178.2 (2)
C4—Zr1—C1—C2	−78.67 (16)	C21—C20—C28—Zr1	−86.5 (2)
C19—Zr1—C1—C2	177.22 (15)	C31—C19—C28—C20	−167.0 (2)
C3—Zr1—C1—C2	−37.42 (14)	Zr1—C19—C28—C20	119.6 (2)
C5—Zr1—C1—C2	−115.8 (2)	C31—C19—C28—C29	11.5 (2)
C31—Zr1—C1—C2	−151.22 (14)	Zr1—C19—C28—C29	−61.97 (14)
C30—Zr1—C1—C2	−143.15 (14)	C31—C19—C28—Zr1	73.46 (13)
C29—Zr1—C1—C2	166.81 (14)	C6—Zr1—C28—C20	1.41 (19)
C28—Zr1—C1—C2	153.82 (14)	Cl1—Zr1—C28—C20	95.19 (19)
C5—C1—C2—C3	0.3 (3)	C4—Zr1—C28—C20	−144.70 (18)
Zr1—C1—C2—C3	65.63 (15)	C19—Zr1—C28—C20	−127.4 (3)
C5—C1—C2—Zr1	−65.38 (16)	C3—Zr1—C28—C20	−122.8 (3)
C6—Zr1—C2—C1	−99.48 (16)	C5—Zr1—C28—C20	−115.01 (19)
Cl1—Zr1—C2—C1	164.82 (14)	C2—Zr1—C28—C20	−56.2 (2)
C4—Zr1—C2—C1	77.19 (16)	C1—Zr1—C28—C20	−79.9 (2)
C19—Zr1—C2—C1	−3.41 (19)	C31—Zr1—C28—C20	−167.6 (2)
C3—Zr1—C2—C1	114.7 (2)	C30—Zr1—C28—C20	151.6 (2)
C5—Zr1—C2—C1	36.41 (14)	C29—Zr1—C28—C20	113.6 (2)
C31—Zr1—C2—C1	38.50 (19)	C6—Zr1—C28—C29	−112.19 (12)
C30—Zr1—C2—C1	77.7 (3)	Cl1—Zr1—C28—C29	−18.41 (12)
C28—Zr1—C2—C1	−42.2 (2)	C4—Zr1—C28—C29	101.70 (14)
C6—Zr1—C2—C3	145.82 (15)	C19—Zr1—C28—C29	119.00 (18)
Cl1—Zr1—C2—C3	50.11 (14)	C3—Zr1—C28—C29	123.5 (3)
C4—Zr1—C2—C3	−37.51 (14)	C5—Zr1—C28—C29	131.39 (13)
C19—Zr1—C2—C3	−118.12 (15)	C2—Zr1—C28—C29	−169.84 (13)
C5—Zr1—C2—C3	−78.29 (15)	C1—Zr1—C28—C29	166.51 (12)
C1—Zr1—C2—C3	−114.7 (2)	C31—Zr1—C28—C29	78.83 (13)
C31—Zr1—C2—C3	−76.21 (18)	C30—Zr1—C28—C29	38.00 (11)
C30—Zr1—C2—C3	−37.0 (3)	C6—Zr1—C28—C19	128.81 (14)
C28—Zr1—C2—C3	−156.90 (13)	Cl1—Zr1—C28—C19	−137.42 (12)
C1—C2—C3—C4	−1.0 (2)	C4—Zr1—C28—C19	−17.31 (17)
Zr1—C2—C3—C4	65.55 (15)	C3—Zr1—C28—C19	4.5 (4)
C1—C2—C3—Zr1	−66.56 (15)	C5—Zr1—C28—C19	12.39 (15)
C6—Zr1—C3—C2	−35.61 (16)	C2—Zr1—C28—C19	71.16 (17)
Cl1—Zr1—C3—C2	−129.94 (15)	C1—Zr1—C28—C19	47.51 (15)
C4—Zr1—C3—C2	114.8 (2)	C31—Zr1—C28—C19	−40.18 (12)
C19—Zr1—C3—C2	89.24 (17)	C30—Zr1—C28—C19	−81.01 (13)
C5—Zr1—C3—C2	76.88 (16)	C29—Zr1—C28—C19	−119.00 (18)
C1—Zr1—C3—C2	36.89 (14)	C22—C23—C29—C28	3.6 (3)
C31—Zr1—C3—C2	124.87 (15)	C22—C23—C29—C30	−170.9 (2)
C30—Zr1—C3—C2	161.63 (14)	C22—C23—C29—Zr1	94.3 (2)
C29—Zr1—C3—C2	−173.21 (15)	C20—C28—C29—C23	−5.7 (3)
C28—Zr1—C3—C2	85.8 (4)	C19—C28—C29—C23	175.60 (18)
C6—Zr1—C3—C4	−150.38 (14)	Zr1—C28—C29—C23	117.56 (18)
Cl1—Zr1—C3—C4	115.29 (15)	C20—C28—C29—C30	170.04 (17)
C19—Zr1—C3—C4	−25.5 (2)	C19—C28—C29—C30	−8.7 (2)
C5—Zr1—C3—C4	−37.89 (14)	Zr1—C28—C29—C30	−66.71 (13)
C2—Zr1—C3—C4	−114.8 (2)	C20—C28—C29—Zr1	−123.25 (17)
C1—Zr1—C3—C4	−77.87 (16)	C19—C28—C29—Zr1	58.04 (14)
C31—Zr1—C3—C4	10.10 (17)	C6—Zr1—C29—C23	−51.57 (17)
C30—Zr1—C3—C4	46.87 (17)	Cl1—Zr1—C29—C23	45.42 (16)
C29—Zr1—C3—C4	72.0 (2)	C4—Zr1—C29—C23	133.18 (17)
C28—Zr1—C3—C4	−28.9 (4)	C19—Zr1—C29—C23	−152.6 (2)
C2—C3—C4—C5	1.4 (2)	C3—Zr1—C29—C23	88.4 (2)
Zr1—C3—C4—C5	68.33 (15)	C5—Zr1—C29—C23	177.32 (16)
C2—C3—C4—Zr1	−66.94 (15)	C1—Zr1—C29—C23	−139.79 (18)
C6—Zr1—C4—C5	−71.66 (18)	C31—Zr1—C29—C23	166.2 (2)
Cl1—Zr1—C4—C5	−176.38 (14)	C30—Zr1—C29—C23	128.9 (2)
C19—Zr1—C4—C5	48.18 (16)	C28—Zr1—C29—C23	−117.2 (2)
C3—Zr1—C4—C5	−113.3 (2)	C6—Zr1—C29—C28	65.66 (12)
C2—Zr1—C4—C5	−76.30 (16)	Cl1—Zr1—C29—C28	162.65 (12)
C1—Zr1—C4—C5	−36.36 (14)	C4—Zr1—C29—C28	−109.60 (14)
C31—Zr1—C4—C5	75.67 (15)	C19—Zr1—C29—C28	−35.39 (12)
C30—Zr1—C4—C5	103.94 (15)	C3—Zr1—C29—C28	−154.35 (15)
C29—Zr1—C4—C5	101.64 (16)	C5—Zr1—C29—C28	−65.46 (15)
C28—Zr1—C4—C5	57.65 (18)	C1—Zr1—C29—C28	−22.6 (2)
C6—Zr1—C4—C3	41.64 (19)	C31—Zr1—C29—C28	−76.56 (13)
Cl1—Zr1—C4—C3	−63.08 (14)	C30—Zr1—C29—C28	−113.83 (17)
C19—Zr1—C4—C3	161.48 (14)	C6—Zr1—C29—C30	179.49 (12)
C5—Zr1—C4—C3	113.3 (2)	Cl1—Zr1—C29—C30	−83.52 (11)
C2—Zr1—C4—C3	37.00 (15)	C4—Zr1—C29—C30	4.24 (16)
C1—Zr1—C4—C3	76.95 (16)	C19—Zr1—C29—C30	78.44 (13)
C31—Zr1—C4—C3	−171.02 (15)	C3—Zr1—C29—C30	−40.5 (2)
C30—Zr1—C4—C3	−142.76 (14)	C5—Zr1—C29—C30	48.38 (16)
C29—Zr1—C4—C3	−145.06 (14)	C1—Zr1—C29—C30	91.27 (18)
C28—Zr1—C4—C3	170.95 (13)	C31—Zr1—C29—C30	37.27 (12)
C2—C1—C5—C4	0.6 (3)	C28—Zr1—C29—C30	113.83 (17)
Zr1—C1—C5—C4	−65.13 (15)	C25—C24—C30—C31	−2.1 (3)
C2—C1—C5—Zr1	65.75 (16)	C25—C24—C30—C29	171.7 (2)
C3—C4—C5—C1	−1.2 (2)	C25—C24—C30—Zr1	−90.0 (2)
Zr1—C4—C5—C1	66.76 (16)	C23—C29—C30—C24	3.1 (4)
C3—C4—C5—Zr1	−68.00 (15)	C28—C29—C30—C24	−171.9 (2)
C6—Zr1—C5—C1	14.76 (17)	Zr1—C29—C30—C24	119.9 (2)
Cl1—Zr1—C5—C1	−110.94 (14)	C23—C29—C30—C31	177.6 (2)
C4—Zr1—C5—C1	−115.5 (2)	C28—C29—C30—C31	2.5 (2)
C19—Zr1—C5—C1	111.70 (15)	Zr1—C29—C30—C31	−65.62 (14)
C3—Zr1—C5—C1	−77.23 (16)	C23—C29—C30—Zr1	−116.8 (2)
C2—Zr1—C5—C1	−36.53 (15)	C28—C29—C30—Zr1	68.11 (13)
C31—Zr1—C5—C1	145.06 (16)	C6—Zr1—C30—C24	−130.72 (17)
C30—Zr1—C5—C1	160.57 (14)	Cl1—Zr1—C30—C24	−39.38 (16)
C29—Zr1—C5—C1	136.00 (14)	C4—Zr1—C30—C24	53.06 (18)
C28—Zr1—C5—C1	105.00 (15)	C19—Zr1—C30—C24	152.2 (2)
C6—Zr1—C5—C4	130.21 (14)	C3—Zr1—C30—C24	29.3 (2)
Cl1—Zr1—C5—C4	4.51 (17)	C5—Zr1—C30—C24	86.14 (18)
C19—Zr1—C5—C4	−132.85 (15)	C2—Zr1—C30—C24	52.6 (3)
C3—Zr1—C5—C4	38.22 (14)	C1—Zr1—C30—C24	100.80 (19)
C2—Zr1—C5—C4	78.92 (15)	C31—Zr1—C30—C24	115.6 (2)
C1—Zr1—C5—C4	115.5 (2)	C29—Zr1—C30—C24	−130.1 (2)
C31—Zr1—C5—C4	−99.48 (15)	C28—Zr1—C30—C24	−167.1 (2)
C30—Zr1—C5—C4	−83.98 (15)	C6—Zr1—C30—C31	113.67 (12)
C29—Zr1—C5—C4	−108.55 (14)	Cl1—Zr1—C30—C31	−155.00 (12)
C28—Zr1—C5—C4	−139.55 (13)	C4—Zr1—C30—C31	−62.55 (13)
Cl1—Zr1—C6—C15	162.55 (13)	C19—Zr1—C30—C31	36.62 (12)
C4—Zr1—C6—C15	58.99 (18)	C3—Zr1—C30—C31	−86.36 (14)
C19—Zr1—C6—C15	−62.03 (15)	C5—Zr1—C30—C31	−29.47 (14)
C3—Zr1—C6—C15	81.28 (15)	C2—Zr1—C30—C31	−63.0 (2)
C5—Zr1—C6—C15	23.42 (16)	C1—Zr1—C30—C31	−14.81 (17)
C2—Zr1—C6—C15	62.80 (15)	C29—Zr1—C30—C31	114.28 (17)
C1—Zr1—C6—C15	31.18 (14)	C28—Zr1—C30—C31	77.33 (13)
C31—Zr1—C6—C15	−75.65 (16)	C6—Zr1—C30—C29	−0.61 (15)
C30—Zr1—C6—C15	−115.76 (14)	Cl1—Zr1—C30—C29	90.72 (11)
C29—Zr1—C6—C15	−116.09 (14)	C4—Zr1—C30—C29	−176.83 (12)
C28—Zr1—C6—C15	−87.23 (14)	C19—Zr1—C30—C29	−77.66 (13)
Cl1—Zr1—C6—C18	44.97 (15)	C3—Zr1—C30—C29	159.36 (12)
C4—Zr1—C6—C18	−58.59 (19)	C5—Zr1—C30—C29	−143.75 (12)
C19—Zr1—C6—C18	−179.61 (15)	C2—Zr1—C30—C29	−177.28 (18)
C3—Zr1—C6—C18	−36.31 (16)	C1—Zr1—C30—C29	−129.09 (13)
C5—Zr1—C6—C18	−94.16 (16)	C31—Zr1—C30—C29	−114.28 (17)
C2—Zr1—C6—C18	−54.78 (16)	C28—Zr1—C30—C29	−36.95 (11)
C1—Zr1—C6—C18	−86.40 (16)	C26—C27—C31—C30	0.9 (3)
C31—Zr1—C6—C18	166.76 (13)	C26—C27—C31—C19	−177.6 (2)
C30—Zr1—C6—C18	126.66 (14)	C26—C27—C31—Zr1	91.2 (2)
C29—Zr1—C6—C18	126.33 (15)	C24—C30—C31—C27	1.0 (3)
C28—Zr1—C6—C18	155.19 (16)	C29—C30—C31—C27	−174.14 (17)
C15—C7—C8—C9	0.5 (3)	Zr1—C30—C31—C27	118.90 (18)
C7—C8—C9—C10	−0.3 (3)	C24—C30—C31—C19	179.87 (18)
C8—C9—C10—C16	−0.3 (3)	C29—C30—C31—C19	4.7 (2)
C17—C11—C12—C13	−0.3 (3)	Zr1—C30—C31—C19	−62.27 (14)
C11—C12—C13—C14	1.6 (3)	C24—C30—C31—Zr1	−117.86 (18)
C12—C13—C14—C18	−0.5 (3)	C29—C30—C31—Zr1	66.96 (13)
C8—C7—C15—C16	0.0 (3)	C28—C19—C31—C27	168.7 (2)
C8—C7—C15—C6	−175.5 (2)	Zr1—C19—C31—C27	−115.6 (2)
C18—C6—C15—C7	−178.3 (2)	C28—C19—C31—C30	−9.9 (2)
Zr1—C6—C15—C7	57.3 (3)	Zr1—C19—C31—C30	65.83 (14)
C18—C6—C15—C16	5.9 (2)	C28—C19—C31—Zr1	−75.78 (13)
Zr1—C6—C15—C16	−118.47 (15)	C6—Zr1—C31—C27	153.34 (17)
C9—C10—C16—C15	0.8 (3)	Cl1—Zr1—C31—C27	−89.24 (18)
C9—C10—C16—C17	−179.7 (2)	C4—Zr1—C31—C27	3.73 (19)
C7—C15—C16—C10	−0.6 (3)	C19—Zr1—C31—C27	128.1 (2)
C6—C15—C16—C10	175.82 (18)	C3—Zr1—C31—C27	−1.8 (2)
C7—C15—C16—C17	179.76 (18)	C5—Zr1—C31—C27	36.02 (19)
C6—C15—C16—C17	−3.8 (2)	C2—Zr1—C31—C27	34.3 (2)
C12—C11—C17—C18	−2.1 (3)	C1—Zr1—C31—C27	54.3 (2)
C12—C11—C17—C16	176.5 (2)	C30—Zr1—C31—C27	−115.1 (2)
C10—C16—C17—C11	1.6 (4)	C29—Zr1—C31—C27	−152.6 (2)
C15—C16—C17—C11	−178.8 (2)	C28—Zr1—C31—C27	167.7 (2)
C10—C16—C17—C18	−179.6 (2)	C6—Zr1—C31—C30	−91.52 (14)
C15—C16—C17—C18	−0.1 (2)	Cl1—Zr1—C31—C30	25.90 (12)
C13—C14—C18—C17	−1.9 (3)	C4—Zr1—C31—C30	118.88 (13)
C13—C14—C18—C6	178.66 (19)	C19—Zr1—C31—C30	−116.76 (17)
C11—C17—C18—C14	3.2 (3)	C3—Zr1—C31—C30	113.38 (13)
C16—C17—C18—C14	−175.69 (18)	C5—Zr1—C31—C30	151.16 (13)
C11—C17—C18—C6	−177.20 (18)	C2—Zr1—C31—C30	149.45 (13)
C16—C17—C18—C6	3.9 (2)	C1—Zr1—C31—C30	169.41 (12)
C15—C6—C18—C14	173.6 (2)	C29—Zr1—C31—C30	−37.43 (11)
Zr1—C6—C18—C14	−64.1 (3)	C28—Zr1—C31—C30	−77.13 (13)
C15—C6—C18—C17	−5.9 (2)	C6—Zr1—C31—C19	25.23 (16)
Zr1—C6—C18—C17	116.40 (16)	Cl1—Zr1—C31—C19	142.66 (11)
C6—Zr1—C19—C31	−160.89 (12)	C4—Zr1—C31—C19	−124.37 (13)
Cl1—Zr1—C19—C31	−51.17 (14)	C3—Zr1—C31—C19	−129.86 (13)
C4—Zr1—C19—C31	57.76 (13)	C5—Zr1—C31—C19	−92.08 (13)
C3—Zr1—C19—C31	71.70 (16)	C2—Zr1—C31—C19	−93.79 (15)
C5—Zr1—C19—C31	81.77 (13)	C1—Zr1—C31—C19	−73.83 (13)
C2—Zr1—C19—C31	113.11 (13)	C30—Zr1—C31—C19	116.76 (17)
C1—Zr1—C19—C31	111.30 (13)	C29—Zr1—C31—C19	79.33 (13)
C30—Zr1—C19—C31	−35.67 (11)	C28—Zr1—C31—C19	39.63 (12)
C29—Zr1—C19—C31	−75.61 (13)	C37—C32—C33—C34	0.3 (3)
C28—Zr1—C19—C31	−109.75 (18)	C38—C32—C33—C34	−179.0 (2)
C6—Zr1—C19—C28	−51.14 (13)	C32—C33—C34—C35	−1.0 (4)
Cl1—Zr1—C19—C28	58.58 (14)	C33—C34—C35—C36	1.3 (4)
C4—Zr1—C19—C28	167.51 (12)	C34—C35—C36—C37	−1.0 (4)
C3—Zr1—C19—C28	−178.54 (13)	C35—C36—C37—C32	0.3 (4)
C5—Zr1—C19—C28	−168.48 (14)	C33—C32—C37—C36	0.1 (3)
C2—Zr1—C19—C28	−137.14 (12)	C38—C32—C37—C36	179.4 (2)
